# Comparative Study of Growth, Cadmium Accumulation and Tolerance of Three Chickpea (*Cicer arietinum* L.) Cultivars

**DOI:** 10.3390/plants9030310

**Published:** 2020-03-01

**Authors:** Shakir Ullah, Jafar Khan, Khizar Hayat, Ahmed Abdelfattah Elateeq, Uzma Salam, Bofan Yu, Yuehua Ma, Hongzheng Wang, Zhong-Hua Tang

**Affiliations:** 1 Key Laboratory of Plant Ecology, Northeast Forestry University, Harbin 150040, China; shakirshamas321@gmail.com (S.U.); qw4133881@nefu.edu.cn (J.K.); khizarhayatnefu637@gmail.com (K.H.); ahmedelateeq@azhar.edu.eg (A.A.E.); uzmashakir321@gmail.com (U.S.); ybfan0903@163.com (B.Y.); myhhgc@163.com (Y.M.); 2 Horticulture Department, Faculty of Agriculture, Al-Azhar University, Nasr City, Cairo 11651, Egypt

**Keywords:** chickpea, cadmium, ICP-OES, growth, physiological responses, tolerance

## Abstract

Trace metals (TM) contamination is a severe problem in the environment and produced an adverse effect on the productivity of crops. Cadmium (Cd) is a TM ranked seven among the top 20 pollutants due to its high toxicity and solubility in water, taken up by the plants and affects their growth and metabolism. In this study, we evaluated the growth, Cd accumulation and tolerance capacities of three chickpea (*Cicer arietinum* L.) cultivars (NC234 (NC2), ICCV89310 (IC8) and ICCV89323-B (IC8-B)), subjected to two Cd concentrations (25 and 50 µM) in hydroponic culture. The toxicity of Cd reduced the plant height and fresh and dry biomass in all cultivars. The maximum reduction was observed at 50 µM of Cd. Compared with IC8-B, cultivars IC8 and NC2 exhibited better performance with high growth, biomass, root to shoot (R/S) ratio and water content under high Cd stress. To measure the accumulation of Cd in root and shoot, an inductively coupled plasma optical emission spectrometer (ICP-OES) was used. IC8 and NC2 had comparatively high Cd tolerance and accumulation ability (> 100 µg g^−1^ dry weight), with IC8 being more tolerant and accumulated higher Cd in shoot than NC2, while cultivar IC8-B was sensitive. Root accumulated more Cd than shoot in a dose-dependent manner. The bioconcentration factors (BCF) and bioaccumulation coefficients (BAC) were far higher than one (> 1) and increased with an increase in Cd concentrations, while the translocation factor (TF) was less than one (< 1), suggesting that all the three cultivars were unable to transfer Cd from the root to the shoot efficiently. Our results indicated that IC8 and NC2 proved to be resistant, while IC8-B showed sensitivity when exposed to high Cd stress (50 µM).

## 1. Introduction

Soil contamination with trace metals (TM), caused by industrial sewage and agricultural production, has become a worldwide problem [1]. The contamination of TM negatively affects the environment as well as decreases agricultural productivity and eventually causing a serious health risk to the consumers [2,3]. Through many natural processes, the contamination of soil is determined, especially by anthropogenic, lithogenic and pedogenic factors [4]. Plants absorb and accumulate TM, such as Cu, Mn, Fe and Zn from the soil, which is required for the normal growth and development at trace level since they are the catalytic and structural components of some enzymes and proteins [5,6]. In contrast, TM (Al, Cr, Pb and Cd) and even those essential for growth and metabolism, may affect different metabolic and physiological processes at high concentrations, such as hindering of functional groups of essential molecules, e.g., enzymes, polynucleotides, transport systems for essential ions and nutrients, the substitution of important ions from cellular sites, inactivation and denaturation of enzymes and distraction of cell and organelle membrane integrity [7]. Among toxic TM, Cd is one of the most mobile non-recyclable pollutants, which is taken up by plants easily and distributed to the above-ground parts where it can accumulate to high levels. Thereby, it can easily enter the food chain [8] and become very detrimental to humans as well as animal’s health [9].

The accumulation of Cd greatly varies amongst plant species and cultivars [10]. Root accumulates a higher amount of Cd than shoot [11]. Several studies revealed that Cd might hamper with different chemical processes such as impregnation through ammonia, compounds of nitrogen and different microbial contact and distresses yield of the plants [12]. According to Ahmad et al. [13], the accumulation of Cd brings complex changes in plants at physiological, biochemical and genetic levels. In plants, Cd is not an essential nutrient element and caused toxic effects at high concentrations. Excessive Cd produces free radicals and reactive oxygen species (ROS), which can oxidize proteins, lipids, DNA and carbohydrates, hence disturbing some physical and biological processes in plants [14]. Toxic effects of Cd are shown in inhibition of photosynthesis and oxidative stress leading to membrane damage as well as altered cellular metabolism [15]. Likewise, the presence of extreme amounts of Cd in the environment causes a variety of plant responses, such as stunted growth, leaf chlorosis, reduced plant fresh and dry biomass and even death [16,17]. Moreover, an excessive amount of Cd may cause inhibition of various enzymes activities and decreased the uptake of nutrient elements (Fe, Cu, Zn and Mn), which can weaken the passage of these elements from roots to the above-ground organs, thus generated to reduce the photosynthetic pigments [18,19]. The primary site of Cd toxicity is unknown; however, the mitochondrial electron transfer chain of plant cells is expected to be one of the major targets of Cd toxicity and is the site of the most rapid Cd-induced ROS production [20].

Underlying the toxic effects of Cd, plants adopt different tolerance mechanisms which include active exclusion, vacuolar restoration, retaining in the roots and immobilization of cell walls and complexation by binding metal to low molecular-weight proteins [21,22]. It has been shown that the decreasing of Cd accumulation by exclusion in roots of *Thlaspi arvense* consults greater tolerance in the Cd-tolerance ecotype [23]. One persistent mechanism for TM metals detoxification within plants is chelation by a ligand. In wheat, Cd binds to the Cd–phytochelatins complexes (Cd–PCs), reducing free Cd^2+^ in the cytosol, and the Cd–PCs complexes are in turn transported into the vacuole or out of the cell by ATP binding cassette transporters [24]. The retention of Cd in the cell wall of the roots is another mechanism of tolerance which might be performed by the carboxyl groups of cell wall proteins or the thiols groups of soluble proteins and non-protein thiols in root cells [25]. Studies have shown that vacuolar compartmentalization prevents the free moments of Cd ions in the cytoplasm and restricts them to limited areas [26]. Besides, transcriptomic in combination with genetic approaches as different to physiological and biochemical studies, may help in understanding the mechanism of metal tolerance. Such as tolerant species in comparison with non-tolerant ones revealed high expression of genes, e.g., *HMA3* might play a role in vacuolar sequestration and metals detoxification [27]. Furthermore, studies have shown that vitamin E (alpha-tocopherol), an important antioxidant, is crucial to oxidative stress tolerance induced by Cd in *Arabidopsis thaliana*, while a chromatin remodeling factor, named *OXS3*, is recognized for Cd tolerance of *Brassica juncea* cDNA library in *Schizosaccharomyces pombe* [20].

Chickpea (*Cicer arietinum* L., family: Fabaceae) is an important pulse crop amongst the legume crops and ranked third after beans and pea plants, which surpasses the production of 8.40 million tons/year in the world [28]. Chickpea is a rich and cheap source of protein, fats and carbohydrates in developing countries and used as a green manure and fodder for animals worldwide [13]. Earlier studies revealed that legume crops are less resistant to Cd toxicity than cereals and grasses and encounter severe suppression of biomass production even at very low levels of Cd [29]. Though, compared with other species, little information is available concerning the ability of tolerance and accumulation in chickpea cultivars under Cd stress. Therefore, the present work has been conducted to screen the chickpea cultivars under Cd stress. The main objectives of the present experiment include: (1) to understand the effect of Cd on growth, and Cd accumulation of chickpea cultivars, (2) to understand differential responses of Cd stress among different chickpea cultivars and (3) to screen Cd-tolerant and non-tolerant chickpea cultivars for further studies.

## 2. Materials and Methods

### 2.1. Chickpea Seeds and Growth Conditions

The seeds of chickpea cultivars (NC234 (NC2), ICCV89310 (IC8) and ICCV89323-B (IC8-B)), were provided for this study by the Crop Genetic Resources Institute, Xinjiang Academy of Agricultural Science, Urumchi, China.

A pot experiment was conducted in the Key Laboratory of Plant Ecology, Department of Botany, Northeast Forestry University, Harbin, China. The selection of the three cultivars was entirely based on the germination percentage and vigorous growth among ten chickpea cultivars (data not shown).

### 2.2. Plants Materials and Cadmium Treatments

Healthy seeds of chickpea were surface sterilized with 75% ethanol solution for 20 s and transferred to 6% NaOCl for 10 min to make them free from infection followed by washing several times with sterile distilled water. The sterilized seeds were then kept in Petri dishes having some distilled water for 24 h under fluorescent white light in a *germinator*. For further growth, one day old ten surface-sterilized seeds were sown in trays containing vermiculite and watered with distilled water to maintain the moisture content efficiently. After 12 days of growth, uniform seedlings were transferred hydroponically to a 1 L plastic container (6 plants per pot) for more 20 days using 20% of a modified Hoagland’s nutrient solution [30]. The compositions of the modified nutrient solution were (Ca(NO_3_)_2_·4H_2_O, 1180 mg L^−1^; KNO_3_, 505 mg L^−1^; MgSO_4_·7H_2_O, 493 mg L^−1^; NH_4_NO_3_, 80 mg L^−1^; KH_2_PO_4_, 68 mg L^−1^, FeEDTA, 22.5 mg L^−1^; H_3_BO_3_, 2.86 mg L^−1^; MnCl_2_·4H_2_O, 1.81 mg L^−1^; ZnSO_4_·7H_2_O, 0.22 mg L^−1^; H_2_MoO_4_·H_2_O, 0.09 mg L^−1^; Na_2_MoO_4_·2H_2_O, 0.12 mg L^−1^ and CuSO_4_·5H_2_O, 0.051 mg L^−1^).

The growth room was climate-controlled with a temperature ranged 22−25 °C, 70% relative humidity and 14-h photoperiod. The nutrient solution was renewed every four days. On the 20th day of seedling transplanting, Cd was supplemented to the nutrient medium as CdCl_2_ in two concentrations, 25 and 50 µM (Cd1 and Cd2, respectively). Simultaneously, control plants were grown in the same nutrient solution without Cd supplementation. In total, the seedlings remained in the nutrient solution for 25 days, including the Cd stress. Cd concentrations were selected based on previously predicted toxicity in chickpea [17,31].

### 2.3. Measurements of Plant Growth and Cd Accumulation

On the 25th day, the seedlings were collected and soaked with 20 mM Na2-EDTA solution for 15 min, washed with sanitized water 3–4 times to take away Cd on the root surfaces and separated into roots and shoots. The length of roots and shoots along with total plant height was done by the measuring tape. To calculate the fresh (FW) and dry (DW) weights of the biomasses, an automatic weighing scale was used. For DW determination, samples were oven-dried at 120 °C for 20 min and continued for three days in the oven at 60 °C. From individual groups of treatments all the biomasses were assessed.

Subsequently, for biochemical analysis, the oven-dried samples were ground with mortar and pestle into powder. The grinded samples were weighed (0.3 g) and digested with a mixture of acids (HNO_3_ + HClO_4_ in the ratio of 5:1 (v/v)). The concentrations of Cd were then quantified by ICP-OES (Optima-8300 DV; PerkinElmer, Inc., Waltham, MA, USA). At least three times every experiment was repeated.

The accumulation of Cd in plant tissues, total Cd accumulation and distribution proportion of Cd in roots were calculated as follows [22]:

Cd accumulation = biomass (DW) × Cd concentration in plant tissues,
Total Cd accumulation = Cd accumulation in root + Cd accumulation in shoot,

Cadmium distribution proportion in root = Cd accumulation in root/total Cd accumulation, while the translocation factor (TF) of Cd from root to shoot, bioconcentration factor (BCF) and bioaccumulation coefficient (BAC) were determined according to the method of Amin et al. [32]. Where Cd^s^ and Cd^r^ represents the Cd concentration in shoot and root respectively, while Cd^ns^ reveals the Cd concentration in nutrient solution.

TF = Cd^s^/Cd^r^,
BCF = Cd^r^/Cd^ns^,
BAC = Cd^s^/Cd^ns^.

### 2.4. Growth Tolerance Indices

Tolerance index (TI) was calculated separately based on dry plant biomass, containing root biomass, shoot biomass and total plant dry biomass according to Wiszniewska et al. [33] with slight modification. Where TI^r^, TI^s^ and TI^tp^ indicates the tolerance indices of root, shoot and total plant, respectively; Cd^r^, Cd^s^ and Cd^tp^ represents the dry weight of root, shoot and total plant under Cd stress, respectively; while MV^rc^, MV^sc^ and MV^tpc^ reveals mean values of root, shoot and total plant dry weight in control, respectively.

TI^r^ = (Cd^r^/MV^rc^) × 100,
TI^s^ = (Cd^s^/MV^Sc^) × 100,
TI^tp^ = (Cd^tp^/MV^tpc^) × 100.

### 2.5. Shoot Water Content Measurement

Water content (WC) was determined after the Cd stress period. For calculating the WC, the FW of the examined plant shoots were recorded. Subsequently, the samples were oven-dried at 60 °C for 72 h and their DW were determined. WC in the shoot was calculated as follows [34]:

WC = (FW − DW)/FW × 100.

### 2.6. Statistical Analysis

The experiment was accomplished following Completely Randomized Design (CRD) with nine treatments and it was repeated three times under the same condition. The data were subjected to analysis of variance (ANOVA) using COSTAT computer package ver. 6.4 (CoHort software in Monterey, CA, USA). The significance of variances between means was taken using the Duncan’s Multiple Range Test (DMRT) at *p* < 0.05. The values were displayed as the mean ± standard error (SE), (*n* = 3).

## 3. Results

### 3.1. Visual Observation and Cd Toxicity

**Figure 1:**
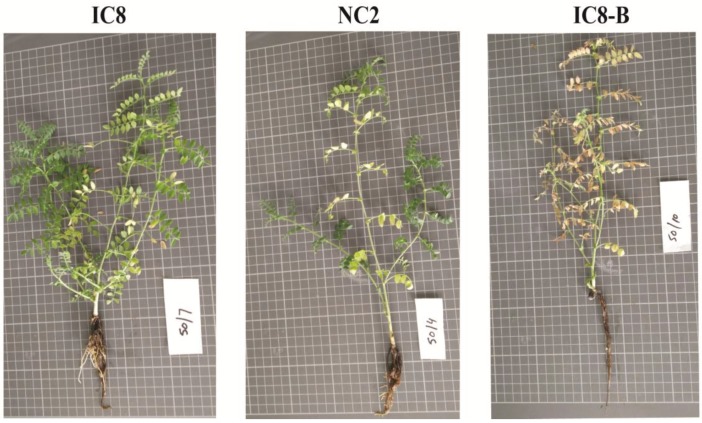
Chlorosis symptoms in hydroponically grown chickpea cultivars (IC8, NC2 and IC8-B) exposed to Cd2 (50 µM) after 25 days of sowing.

Chickpea cultivars NC2, IC8 and IC8-B were subjected to different concentrations of Cd, including control, 25 µM (Cd1) and 50 µM (Cd2). Visual observations showed that the toxic effect was concentration dependent and each cultivar responded differently to the Cd stress in the growth medium. All cultivars showed chlorosis and necrosis at both Cd treatments, although these effects were noticeable to a greater extent in IC8-B cultivar under high Cd stress (50 µM) compared to NC2 and IC8 cultivars (Figure 1). However, at Cd1, the chlorosis caused by Cd toxicity was observed to a smaller extent in all cultivars. Plants without Cd treatment (control) also showed some natural chlorosis. Regarding the health and chlorosis in seedlings, the following phytotoxicity trend was noticed among cultivars: IC8-B > NC2 > IC8.

### 3.2. Plant Growth Measurement

Plant growth parameter was used as one of an important criterion to assess the plant tolerance to Cd stress. The data associated with growth (root and shoot length and plant height) were assembled in Table 1. It could be observed that the increase of Cd concentrations in the nutrient solution induced significant decrease (*p* < 0.05) in heights of the three chickpea cultivars (NC2, IC8 and IC8-B) compared to control. However, no significant difference was noticed between Cd1 and Cd2 for IC8 cultivar. The highest root lengths (10.01 ± 0.43 and 9.91 ± 0.28 cm) were observed for cultivar IC8 growing on Cd1 and Cd2, respectively, which suggest that IC8 showed more tolerance for Cd toxicity than other ones in this respect. A significant decrease in root growth (7.80 ± 0.52 cm) was recorded for IC8-B cultivar upon exposure to Cd2 treatment. The data revealed similar results for shoot length and total plant height, as IC8 showed more resistance while cultivar IC8-B represented less tolerance.

### 3.3. Biomass Production

#### 3.3.1. Fresh Biomass

The fresh biomass of root was decreased without significant changes in all cultivars by increasing Cd stress in the growth medium compared to control, except cultivar IC8-B which exhibited a significant (*p* < 0.05) reduction at Cd2 treatment (Table 2). In contrast, both NC2 and IC8-B cultivars recorded a significant reduction in FW at Cd2 treatment. It was noticed that cultivar IC8 had no significant reduction in shoot FW at Cd1 and Cd2 treatments when compared with its control. Moreover, the results showed that cultivar IC8 had less fresh biomass reduction under Cd stress, especially at high Cd treatment (50 µM) compared to NC2 and IC8-B cultivars, suggesting their strong resistivity and tolerance to the examined levels of Cd.

#### 3.3.2. Dry Biomass

Dry biomass of chickpea cultivars was reduced significantly (*p* < 0.05) for root, shoot and the whole plant in a dose-dependent manner (Table 3). However, no significant decreases were recorded at Cd1 and Cd2 treatments for IC8 cultivar. Moreover, a significant difference (*p* < 0.05) was also observed between IC8 and IC8-B at Cd2 concentration, but not for NC2 cultivar. Under high Cd stress (50 µM), IC8 cultivar accumulated higher dry biomass, i.e., 0.24 ± 0.05, 1.05 ± 0.21 and 1.30 ± 0.26 g for root, shoot and the whole plant, respectively, while IC8-B exhibited lower dry biomass under the same Cd level. However, a comparatively higher root, shoot and whole-plant dry biomass (0.30 ± 0.03, 1.22 ± 0.05 and 1.52 ± 0.09 g, respectively) were recorded for IC8-B compared with NC2 and IC8 cultivars grown in a nutritive solution containing 25 µM of Cd. Hence, IC8 cultivar could be considered more tolerant than IC8-B at 50 µM of Cd.

### 3.4. Shoot Water Content (WC)

**Figure 2:**
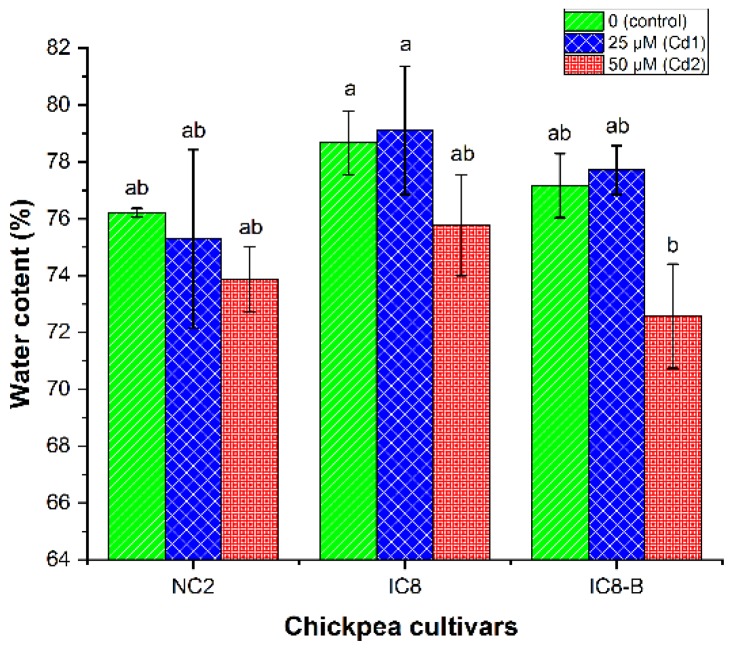
The shoot water content of chickpea cultivars (NC2, IC8 and IC8-B) exposed to various concentrations of Cd (0, 25 and 50 µM). The bars represent SE, *n* = 3. Columns with different characters are significantly different according to (DMRT) at *p* < 0.05.

Shoot water content (WC) of all chickpea cultivars decreased with the increase of Cd in nutrient solution compared to their controls; however, a slight increase was observed in IC8 and IC8-B cultivars at Cd1 stress (Figure 2). The decrease in the WC of shoot between cultivars and their corresponding controls was not significant. Under Cd stress, IC8 contained high WC in the shoot at both treatments compared with the other cultivars, while the lowest WC was noticed for cultivar IC8-B at Cd2 concentration. The demonstrated findings revealed that high Cd stress disturbs the water plant relationship in chickpea cultivars.

### 3.5. Biomass and Cd Roots/Shoot (R/S) Ratio

**Figure 3:**
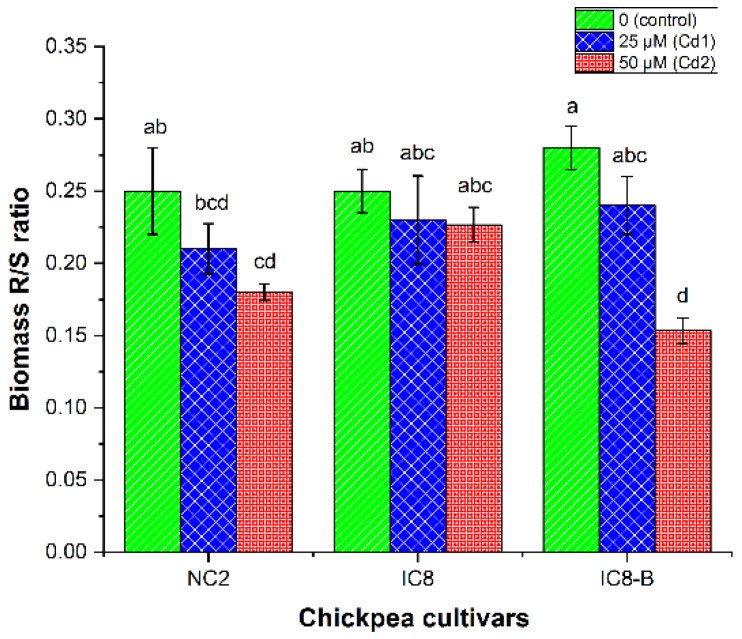
Biomass R/S ratio of chickpea cultivars (NC2, IC8 and IC8-B) exposed to various concentrations of Cd (0, 25 and 50 µM). The bars represent SE, *n* = 3. Columns with different characters are significantly different according to (DMRT) at *p* < 0.05.

**Figure 4:**
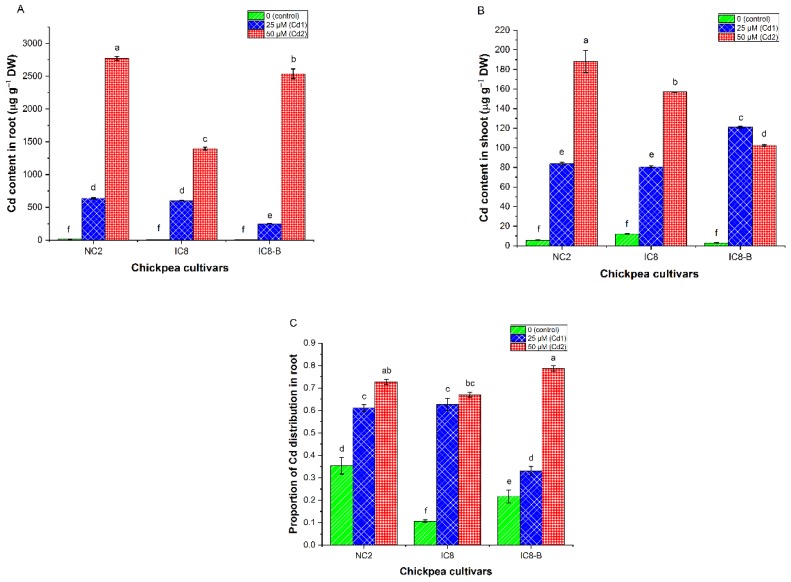
Cadmium content in the root (**A**), shoot (**B**), and distribution proportion in the root (**C**) of chickpea cultivars (NC2, IC8 and IC8-B) under various concentrations of Cd (0, 25 and 50 µM). The bars represent SE, *n* = 3. Columns with different characters are significantly different according to (DMRT) at *p* < 0.05.

The ratio of biomass of the root to the shoot (R/S) was measured based on the DW of the plants. The biomass of the R/S ratio of chickpea cultivars was decreased by increasing Cd concentration in the nutrient solution (Figure 3). Cultivar IC8-B at lower Cd stress (25 µM) had a high biomass R/S ratio (0.24 ± 0.02) compared to NC2 and IC8 cultivars without significant difference. At higher Cd stress (50 µM), cultivar IC8 recorded the highest biomass ratio of R/S (0.23 ± 0.01). Furthermore, our findings showed that the increasing concentration of Cd reduced biomass R/S ratio of NC2 and IC8-B cultivars, while no substantial changes were noticed in cultivar IC8 compared to their controls. However, IC8-B at high Cd stress (50 µM) showed the lowest biomass of the root to shoot (R/S) ratio than IC8 and NC2 cultivars. On the other hand, Cd R/S ratios of all tested cultivars were significantly enhanced with Cd supplemented to the growth medium (Table 4). The highest Cd R/S ratio (24.74 ± 0.53) was recorded for cultivar IC8-B at Cd2, followed by NC2 (14.82 ± 0.74) and IC8 (8.89 ± 0.14). The highest Cd R/S ratio of cultivar IC8-B is further supported by the higher distribution proportion of Cd in the root (Figure 4C), while the lowest Cd R/S ratio of IC8 may be due to the inadequate amount of Cd in the root (Figure 4A).

### 3.6. Tolerance Indices (TI)

Tolerance indices (TI) for all cultivars were different under Cd stress (Table 5). The increasing supply of Cd concentration in nutrient medium tends to reduce the TI for all cultivars. No significant changes in root tolerance indices (TI^r^) were observed for all cultivars at Cd1, while at Cd2, cultivar IC8 had significantly higher TI^r^ value compared to NC2 and IC8-B cultivars. Similarly, no significant changes in the shoot (TI^s^) and total plant (TI^tp^) tolerance indices were noticed among the three cultivars at Cd1 treatment. However, at Cd2 concentration, IC8 cultivar showed significantly higher TI^s^ and TI^tp^, followed by NC2 and IC8-B cultivars.

For all cultivars, the TI values of root, shoot and total plant were decreased with the increase of Cd stress in the nutrient solution. As shown in Table 5, cultivar IC8 was found to be the most tolerant cultivar followed by NC2 under Cd stress. The TI values for root, shoot and total plant were in the range of 84–80%, 95–86% and 93–85% for IC8 and 74–47%, 91–66% and 88–62% for NC2, respectively, while cultivars IC8-B was found to be less tolerant with TI values for root, shoot and total plant in the range of 80–30%, 93–55% and 90–49%, respectively.

### 3.7. Cadmium Contents and Distribution Proportion

In various parts of the chickpea cultivars, the content of Cd was different (Figure 4A,B). It could be observed that Cd content increased significantly (*p* < 0.05) in root and shoot when the Cd stress increased in the nutritive solution. The results illustrated in Figure 4A showed that root organs of all cultivars retained a greater amount of Cd than shoot. Cultivar NC2 had significantly higher Cd content in root ranged (636.02 ± 10.03–2772.33 ± 29.82 µg g^−1^ DW) compared to cultivars IC8-B (249.96 ± 3.58–2538.41 ± 74.13 µg g^−1^ DW) and IC8 (600.68 ± 3.57 - 1395.60 ± 20.65 µg g^−1^ DW) under Cd stress. In shoot, the contents of Cd in the three cultivars were 188.15 ± 11.44, 156.96 ± 0.26 and 102.56 ± 0.79 µg g^−1^ DW for NC2, IC8 and IC8-B, respectively, under higher stress of Cd (50 µM). Cultivar NC2 and IC8-B had the highest Cd content in shoot at Cd2 and Cd1 treatment, respectively. The above results revealed that cultivar IC8 had a lesser amount of Cd at root level due to translocated the absorbed Cd to the aerial parts (shoot); while in contrast, cultivar IC8-B absorbed more Cd from the nutrient solution but had low flow towards the shoot organ when the Cd concentration in the solution was increased.

The distribution proportion of Cd in root was an important index to assess the ability of Cd transfer in chickpea cultivars. It could be observed that the distribution proportion of Cd in root was concentration-dependent and significantly increased (*p* < 0.05) in all cultivars grown under the two Cd concentrations (Figure 4C). Upon exposure to 50 µM of Cd, cultivar IC8-B showed a higher distribution proportion (0.79 ± 0.01), followed by NC2 and IC8 cultivars. At 25 µM of Cd, NC2 (0.61 ± 0.01) and IC8 (0.62 ± 0.02) showed the highest distribution proportion of Cd in the root, while cultivar IC8-B recorded the lowest value (0.33 ± 0.02). The proportions existed in cultivars NC2, IC8 and IC8-B were in the range of 35–73%, 11–61% and 22–79%, respectively.

### 3.8. Cadmium Accumulation

**Figure 5:**
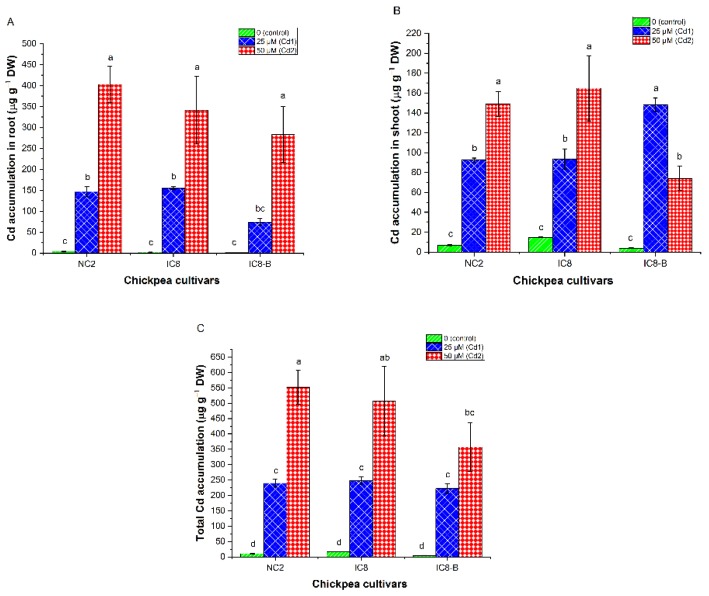
The accumulation of Cd in the root (**A**), shoot (**B**) and total Cd accumulation (**C**) in chickpea cultivars (NC2, IC8 and IC8-B) grown under various concentrations of Cd (0, 25 and 50 µM). The bars represent SE, *n* = 3. Columns with different characters are significantly different according to (DMRT) at *p* < 0.05.

Cd accumulation (µg per plant root, plant shoot or total plant) was calculated by taking into account the Cd content (µg g^−1^ DW) and the dry biomass of root, shoot or total plant (g plant^−1^). Cd accumulation in root and shoot organs of the three chickpea cultivars was observed under the effect of 25 and 50 µM of Cd stress with superior accumulation in root since most Cd was stored in it (Figure 5A,B). Shoot of the three cultivars exhibited a linear increase in Cd accumulation upon exposure to 0, 25 and 50 µM of Cd, except for IC8-B which showed a decline in Cd accumulation at Cd2 (50 µM; Figure 5B). Exposure to Cd1 level cultivar IC8-B exhibited a significantly higher Cd accumulation in shoot than NC2 and IC8 (148.10 ± 6.77, 92.62 ± 1.99, 93.60 ± 10.27 µg per plant shoot, respectively). Excessive stress caused by Cd2 resulted in more accumulation of Cd in IC8 and NC2 than IC8-B cultivar (164.92 ± 32.79, 149.19 ± 12.43 and 74.19 ± 12.16 µg per plant shoot, respectively). Concerning total Cd accumulation, no significant differences were noticed when comparing all cultivars at Cd1. While at Cd2, cultivar NC2 accumulated more total Cd followed by IC8 and IC8-B cultivars (551.96 ± 54.48, 506.20 ± 113.55, 357.33 ± 79.00 µg per total plant, respectively; Figure 5C).

### 3.9. Translocation Factor (TF), Bioconcentration Factor (BCF) and Bioaccumulation Coefficient (BAC)

The translocation factor (TF) was used to measure the ability of a plant to transfer Cd from the root to the shoot. TF is the ratio of Cd content in shoot tissue to that in root one. The TF values of all chickpea cultivars under different Cd treatments are listed in Table 6. Our findings showed that the TF values of all cultivars were significantly changed by increasing Cd levels in the growth medium. The highest TF values were observed for the controlled growth in all cultivars. Upon exposure to Cd1, cultivar IC8-B showed the highest TF values (0.48 ± 0.01), while no significant changes were noticed between NC2 and IC8 cultivars. For Cd2 treatment, no significant differences were recorded among all cultivars.

Using bioconcentration factor (BCF), the ability of a plant to accumulate Cd from nutrient solution in root could be measured. Results showed that BCF was in a dose-dependent manner for all chickpea cultivars and was increased significantly with the increase of Cd stress (Table 6). At Cd2 (50 µM) treatment, cultivar NC2 had a significantly (*p* < 0.05) higher BCF (242.81 ± 2.61) value than cultivars IC8-B (222.33 ± 6.49) and IC8 (122.23 ± 1.80). Besides, to qualify the accumulation of cadmium proficiency in chickpea cultivars, the bioaccumulation coefficient (BAC) must be taken into consideration. Similar to BCF, BAC also increased by increment Cd level in the growth medium. Cultivar IC8 showed the highest BAC (18.32 ± 4.54) followed by NC2 (16.48 ± 1.00) and IC8-B (8.98 ± 0.06) at Cd2 treatment. Furthermore, the results listed in Table 6 showed that the BAC for the tested chickpea cultivars was less than BCF at all treatments (control, 25 and 50 µM) due to higher Cd content in root than shoot organs.

## 4. Discussion

### 4.1. Growth and Biomass Production

Exposure of plants to some heavy or trace elements may lead to toxic effects on growth parameters, such as reduced plant height, root length, FW and DW, although the degree of these negative effects are depended on plant species, genotype and also plant tissue. All these growth attributes are considered very sensitive parameters used as an indicator to measure the response of plants towards metals toxicity [16,28]. Worldwide, agriculture soil is polluted by Cd and causes a greater reduction of plant growth and decreases productivity [35]. In plants, the toxicity of Cd varies with growth conditions and experimental setup and depends on the Cd availability, exposure period and age of plants [29]. In the present study, root and shoot length, plant height, and FW and DW of three chickpea cultivars (NC2, IC8 and IC8-B) were significantly different in response to Cd toxicity. The results presented here revealed that high Cd stress (Cd2; 50 µM) induced greater toxicity and significantly reduced the root and shoot length along with FW and DW of the examined cultivars. Among the studied cultivars, IC8 recorded higher growth, as well as fresh and dry biomass values, compare to NC2 and IC8-B (Table 1), suggesting its tolerance to Cd stress. In contrast, IC8-B had low values for such parameters showing a high sensitivity under Cd stress. The obtained results are consistent with the findings of other authors [10,36]. Rai et al. [37] also reported a significant reduction in root and shoot length as well as in FW and DW in *Phyllanthus amarus* grown under high Cd stress.

The prior indication of Cd toxicity could be measure from visual observation of the plants because it can damage the health of the plant by disrupting the structure of chloroplasts. The most common indication in plants leaves is chlorosis [34,38]. Commonly, the Cd concentration > 5–10 mg Kg^−1^ in leaf tissue is considered poisonous to the plant [39]. In the present study, it was noticed that chlorosis in all cultivars was more apparent than necrosis, although the toxicity was in a dose-dependent manner. At Cd2 (50 µM), IC8-B was more affected and exhibited greater symptoms of chlorosis than NC2 and IC8 cultivars, and thus it could be suggested that IC8-B can be used as a control for the other two cultivars (Figure 1). To some extent, natural chlorosis and necrosis were also observed in all control plants. Concerning visual observations, IC8 seems to be more tolerant than IC8-B under high stress (50 µM). According to Zhang et al. [40], chlorosis in plants grown under the Cd effect may results from the toxic level of Cd by reducing the accumulation of essential elements in the above aerial parts. Changes exhibited in the growth and development and diminishing of visual effects upon exposure to Cd toxicity revealed varietal changes throughout the ontogeny of chickpea cultivars for Cd tolerance. A similar study about chlorosis in certain plant species has been reported by different authors, including chickpea (*Cicer arietinum* L.) [18], *Sedum alfredii* [34], rice (*Oryza sativa* L.) [41] and mungbean (*Vigna radiata*) [42].

### 4.2. Water Relation

Water homeostasis is not only important for the life processes of plants but also the whole living organism on earth. Different environmental abiotic stresses affect the plant tissues by reducing their leaf relative water content [43]. The root is the first site of contact for TM and significantly higher metals content accumulated in them than shoot of the plants, which may be the reason that affected the absorption of water, and in turn, reduce water content in the root [44]. In the shoot, water content was an important parameter to assess the tolerance level of chickpea cultivars under different concentrations of Cd (control, Cd1 and Cd2). Our study showed that shoot WC of all chickpea cultivars decreased at Cd1 and Cd2 of Cd stress without significant changes compared to control seedlings. However, a slight increase in WC was noticed in IC8 and IC8-B cultivars at Cd1 treatment (Figure 2), which also observed in *S. alfredii* when treated with 200 µM Cd [34].

Furthermore, IC8 exhibited a higher amount of WC in shoots than NC2 and IC8-B, proved to be a more tolerant cultivar under high Cd stress (50 µM). In contrast, cultivar IC8-B exhibited less WC in shoots at Cd2 (50 µM) concentration, which could be due to the xylem conductivity reduced by Cd-induced weakening of the cross-sectional area prevailing for water transportation [44] or might be due to the higher quantity of Cd assimilation in root, which can hinder the growth and inhibited the transport of water to the above-ground parts [34,45]. Although, at Cd2, cultivar IC8-B revealed higher reduction in shoot water content, hence, could be suggested more sensitive than IC8 and NC2 cultivars. Consequently, the relationship concerning plant water status and tolerance to Cd had been reported by Belimov et al. [46] in pea mutant SGECd. In plant tissues, the transportation of water is reduced by Cd stress, hence, decreased the transpiration rate, stomatal conductance, WC and resulted in water stress [39,44]. Different researchers have been demonstrated significant changes in RWC under Cd concentrations in various plant cultivars, such as moth bean (*Vigna aconitifolia* L.) [47], bush bean (*Phaseolus vulgaris*) [48] and *Lupinus albus* [49].

### 4.3. Growth Tolerance Indices

Different tolerance indices were measured to examine the tolerance of the three cultivars of chickpea grown under Cd stress. Previously published investigations on the toxicity of Cd in plants reported that Cd caused a reduction in growth and biomass. However, the toxicity was depended on the Cd availability, plant age and the time of exposure [29,50]. To observe the capacity of TM tolerance, an important parameter, i.e., tolerance index (TI), is taken in plants [51]. Our results presented here for tolerance indices of the root (TI^r^), shoot (TI^s^) and total plant (TI^tp^) of chickpea cultivars were significantly different at various Cd treatments and decreased in a dose-dependent manner. TI^s^ were much higher than TI^r^ for all cultivars at both Cd levels, suggesting that shoot biomass was increased while root biomass was reduced under Cd stress, which also agreed with Wiszniewska et al. [33] in *Alyssum montanum* and Barzanti et al. [52] in *A. montanum* and *A. bertolonii*.

Amongst the studied cultivars, IC8 had significantly higher TI^r^, TI^s^ and TI^tp^, followed by NC2 and IC8-B, respectively, at high Cd treatment (50 µM). The deleterious effect of Cd had also been described by Shamsi et al. [53] in soybean genotypes under hydroponic medium. Early studies in legume crops persistently showed a greater inhibition under low Cd stress [54], while conflicting results demonstrated by Metwally et al. [29] in pea genotypes as exhibited greater variation in growth responses from each other under Cd stress. In the present study, the identified TI^r^ and TI^s^ based on dry biomass of chickpea cultivars responded differently under the high stress of Cd. The values of TI for cultivar IC8 were higher than NC2 and IC8-B, suggesting it to be Cd-tolerant. It could be due to Cd-binding protein existence in IC8, which facilitates the growth and photosynthesis of the plants [39]. In contrast, cultivar IC8-B showed sensitivity when exposed to high Cd stress (50 µM). According to Lux et al. [55], plant species with TI > 60 are tolerant.

### 4.4. Biomass and Cadmium R/S Ratio

The ratio of the root to the shoot (R/S) had been received greater attention in the last few decades and used as an indicator to evaluate the overall health of the plant in environmental stress conditions [56]. If a plant has a high R/S ratio, it will absorb more nutrients from their surroundings and thus increase the above-ground biomass as well as increasing the adaptability and tolerance in stress medium. Therefore, to assess the tolerance level of chickpea cultivars (NC2, IC8 and IC8-B), biomass R/S and Cd R/S ratios were taken under different treatments of Cd. The results showed a significant decline in biomass R/S ratio of cultivar NC2 and IC8-B under Cd stress compared to control. However, cultivar IC8 showed no significant changes compared to control and exhibited higher biomass R/S ratio at Cd2 concentration, suggesting their tolerance to Cd in this respect (Figure 3). These findings are agreed with previous studies [16,38].

On the other side, the Cd R/S ratio of the examined cultivars was increased with the increasing of Cd supply in the nutrient solution (Table 4). The R/S ratio of Cd was higher in all cultivars than that of biomass one, which is supported by the findings of Nikolić et al. [57]. In less sensitive (tolerant) genotypes, it was reported that translocation of Cd from the root to the shoot was low, while sensitive genotypes showed high transfer from the root to the shoot [29]. In accordance, our results revealed that cultivar IC8 had a low Cd R/S ratio compared to NC2 and IC8-B, endorsing that cultivar IC8 was tolerant while IC8-B was sensitive to tested Cd stress.

### 4.5. Cadmium Concentration and Distribution Proportion in Chickpea Cultivars

It is important to understand the concentration and distribution proportion of Cd in the root tissues of the studied cultivars, to find out the tolerant and sensitive chickpea cultivars. Several studies have shown that root is the first site of contact and account for a greater amount of TM [58,59]. Mobin et al. [60] showed that like other TM, roots retained a higher concentration of Cd and transferred less to the above-ground parts. Although the content of Cd in IC8 root was lower than NC2 and IC8-B, however, the shoot of IC8 recorded higher Cd content than IC8-B, while less than cultivar NC2 (Figure 4A,B). The variation of Cd content in root and shoot indicates the difference of Cd distribution between chickpea cultivars, and this is consistent with the results of Afzal et al. [10] and Nikolić et al. [57].

The content of Cd in chickpea cultivars and its distribution proportion can be used as parameters to detect stress tolerance in chickpea cultivars. Hence, at high Cd stress (50 µM), cultivar NC2 was considered less tolerant and the suggested control cultivar (IC8-B) was sensitive because higher Cd contents were observed in their root, which may affect the cell wall of the root, signalling mechanism of metabolites and nutritional uptake leading to less above-ground biomass. While in cultivar IC8, the distribution of Cd was higher and more Cd localized in the shoot, which accounts for more above ground-biomass because the Cd content was less and no such damage produced at the root level. Therefore, cultivar IC8 was considered more tolerant due to the high Cd distribution proportion. Various factors may be involved in the retention of Cd in the root, such as a negatively charged surface of the cell wall, chelating in cytosol or compartmentalization in the vacuole, cross-linking of Cd with the carboxyl group of the cell wall protein and the interaction with soluble and non-soluble protein thiol group [61,62]. However, further experimentation must be established to explain the tolerance mechanism amongst cultivars NC2, IC8 and IC8-B under Cd stress.

### 4.6. Cd Uptake and Accumulation Capacities

It has long been recognized that TM accumulation ability remarkably varies amongst different plant species and cultivars and is affected by the soil characteristics, TM accessibility in soil and plant genotypes [8,38,63]. In this study, NC2 accumulated more Cd in the root (Figure 5A), while IC8 exhibited higher Cd accumulation in its shoot (Figure 5B). The obtained results revealed a higher accumulation of Cd in the root than in shoot (Figure 5), which is consistent with the findings of Delpérée et al. [64]. The accumulation of Cd in root and shoot of the examined chickpea cultivars was concentration-dependent. Moreover, cultivar IC8-B showed a reduction in Cd accumulation in root and shoot especially under high Cd stress, which could be attributed to the low flowing of water (low WC, Figure 2), and in turn, the xylem not sapped well. Lefèvre et al. [65] showed that Cd has negative effects on plant water relations, and a limited supply of water decreased the uptake and accumulation of metals. The present study revealed that even though a greater amount of Cd was found in the root, however, still significant amounts were found in the shoot, where Cd accumulation was > 100 µg g^−1^ DW in IC8 and NC2 cultivars. The greater accumulation of Cd in the shoot of IC8 and NC2 together with high shoot biomass production suggests its tolerance against high Cd stress, while cultivar IC8-B was sensitive. The obtained results are in harmony with the results of other authors [10,66], which demonstrated that certain tolerant cultivars accumulate high Cd in the shoot, which is the distinguishing feature of plants having high biomass.

Additionally, to qualify the Cd accumulation in chickpea cultivars, the bioconcentration factor (BCF) and bioaccumulation coefficient (BAC) must be taken into consideration. BCF and BAC are excellent indicators of metals accumulation because it takes into account the ratio of Cd concentration in the growth medium and plant tissues (root and shoot, respectively; Table 6) [67]. The chickpea cultivars studied here had BCF and BAC far higher than one (> 1) and increased in a dose-dependent manner, which is an agreement with the findings of Pernia et al. [68]. The tendency to translocate Cd from the root to the shoot, as estimated by the translocation factor (TF) was less than one (< 1), except in the control media for IC8 and IC8-B, suggesting that all of them were unable to move Cd from the root to the shoot efficiently, which agreed with other findings [38,69].

## 5. Conclusions and Future Prospects

The current study showed that high cadmium (Cd) stress (50 µM) in nutrient solution significantly influenced the physiological and biochemical features of the studied cultivars. It was noticed that in the three chickpea cultivars, the reduction in growth and biomass was due to the uptake and accumulation of Cd and decreased with the increase of Cd stress. Besides, Cd markedly influenced the plant height and had negative effects on plant water relations. Furthermore, our results showed variations in Cd tolerance among the three cultivars. Cultivar IC8 had higher Cd accumulation in shoot than cultivars NC2 and IC8-B because of its high biomass production and effective tolerance strategies, while IC8-B showed more symptoms of chlorosis and necrosis compared to the other one. Better performance in terms of growth and biomass was observed in cultivars IC8 and NC2 than IC8-B under high Cd stress. Therefore, considering the Cd accumulation ability and physiological features, we could conclude that among the studied chickpea cultivars, IC8 and NC2 were more tolerant and IC8-B is more sensitive under Cd stress. Therefore, when assessing the growth and tolerance of other chickpea cultivars, IC8-B could be used as a control cultivar under Cd stress. The intraspecific difference of tolerance and accumulation of Cd exists almost in every plant’s species as designated from the differential response of all the cultivars to Cd toxicity. However, further study, especially in the Cd contaminated field conditions, should be established to investigate the metabolomics and physiological mechanism of tolerant cultivars.

## Figures and Tables

**Table 1:** Root and shoot length, and total plant height of chickpea seedlings grown in a nutrient medium under Cd stress at the rate of 0 µM (control), 25 µM (Cd1) and 50 µM (Cd2) with mean values ± SE, (*n* = 3).

Cultivars	Treatments	Root Length ± SE (cm)	Shoot Length ± SE (cm)	Total Height ± SE (cm)
NC2	control	10.62 ± 0.23 ^bc^	18.48 ± 2.08 ^ab^	29.10 ± 2.15 ^abc^
	Cd1	9.80 ± 0.08 ^cd^	15.65 ± 0.73 ^bcd^	25.45 ± 0.66 ^cde^
	Cd2	9.12 ± 0.32 ^d^	14.08 ± 1.28 ^cd^	23.20 ± 1.36 ^ef^
IC8	control	11.37 ± 0.31 ^b^	20.38 ± 1.61 ^a^	31.74 ± 1.86 ^a^
	Cd1	10.01 ± 0.43 ^cd^	17.93 ± 0.41 ^abc^	27.94 ± 0.60 ^abcd^
	Cd2	9.91 ± 0.28 ^cd^	16.40 ± 0.70 ^bcd^	26.31 ± 0.88 ^bcde^
IC8-B	control	12.62 ± 0.62 ^a^	17.60 ± 0.57 ^abc^	30.22 ± 1.20 ^ab^
	Cd1	9.15 ± 0.46 ^d^	15.17 ± 1.37 ^bcd^	24.31 ± 1.74 ^def^
	Cd2	7.80 ± 0.52 ^e^	13.26 ± 0.47 ^d^	21.06 ± 0.86 ^f^

Different characters in the column indicate significant difference and the same characters indicate insignificance according to the Duncan’s Multiple Range Test (DMRT) at *p* < 0.05.

**Table 2:** Fresh biomasses of the roots, shoots and total plant of chickpea seedlings grown in a nutrient medium under Cd stress at the rate of 0 µM (control), 25 µM (Cd1) and 50 µM (Cd2) with mean values ± SE, (*n* = 3).

Cultivars	Treatments	Root Fresh Biomass ± SE (g/plant)	Shoot Fresh Biomass ± SE (g/plant)	Whole Plant Fresh Biomass ± SE (g/plant)
NC2	control	1.41 ± 0.41 ^ab^	5.10 ± 0.45 ^a^	6.51 ± 0.85 ^ab^
	Cd1	1.33 ± 0.01 ^ab^	4.62 ± 0.54 ^a^	5.95 ± 0.54 ^ab^
	Cd2	1.24 ± 0.15 ^ab^	3.07 ± 0.35 ^bc^	4.32 ± 0.50 ^bc^
IC8	control	1.97 ± 0.43 ^a^	5.76 ± 0.38 ^a^	7.73 ± 0.29 ^a^
	Cd1	1.91 ± 0.48 ^ab^	5.55 ± 0.06 ^a^	7.47 ± 0.42 ^a^
	Cd2	1.90 ± 0.50 ^ab^	4.43 ± 1.03 ^ab^	6.33 ± 1.53 ^ab^
IC8-B	control	2.01 ± 0.32 ^a^	5.80 ± 0.19 ^a^	7.81 ± 0.50 ^a^
	Cd1	1.91 ± 0.18 ^ab^	5.48 ± 0.10 ^a^	7.40 ± 0.23 ^a^
	Cd2	0.80 ± 0.20 ^b^	2.65 ± 0.42 ^c^	3.45 ± 0.55 ^c^

Different characters in the column indicate significant difference and the same characters indicate insignificance according to (DMRT) at *p* < 0.05.

**Table 3:** Dry biomasses of the root, shoot and total plant of chickpea seedlings grown in a nutrient medium under Cd stress at the rate of 0 µM (control), 25 µM (Cd1) and 50 µM (Cd2) with mean values ± SE, (*n* = 3).

Cultivars	Treatments	Root Dry Biomass ± SE (g/plant)	Shoot Dry Biomass ± SE (g/plant)	Whole-Plant Dry Biomass ± SE (g/plant)
NC2	control	0.31 ± 0.06 ^ab^	1.21 ± 0.11 ^a^	1.53 ± 0.17 ^a^
	Cd1	0.23 ± 0.01 ^bc^	1.11 ± 0.01 ^ab^	1.34 ± 0.02 ^ab^
	Cd2	0.15 ± 0.01 ^cd^	0.79 ± 0.06 ^bc^	0.94 ± 0.08 ^bc^
IC8	control	0.31 ± 0.01 ^ab^	1.22 ± 0.01 ^a^	1.52 ± 0.03 ^a^
	Cd1	0.26 ± 0.01 ^b^	1.17 ± 0.13 ^a^	1.42 ± 0.13 ^a^
	Cd2	0.24 ± 0.05 ^bc^	1.05 ± 0.21 ^ab^	1.30 ± 0.26 ^ab^
IC8-B	control	0.37 ± 0.02 ^a^	1.32 ± 0.02 ^a^	1.69 ± 0.04 ^a^
	Cd1	0.30 ± 0.03 ^ab^	1.22 ± 0.05 ^a^	1.52 ± 0.09 ^a^
	Cd2	0.11 ± 0.02 ^d^	0.72 ± 0.11 ^c^	0.83 ± 0.13 ^c^

Different characters in the column indicate significant difference and the same characters indicate insignificance according to (DMRT) at *p* < 0.05.

**Table 4:** Cadmium root/shoot (R/S) ratio of chickpea seedlings grown in a nutrient medium under Cd stress at the rate of 0 µM (control), 25 µM (Cd1) and 50 µM (Cd2) with mean values ± SE, (*n* = 3).

Treatments	NC2	IC8	IC8-B
control	2.18 ± 0.09 ^e^	0.50 ± 0.01 ^f^	0.99 ± 0.16 ^f^
Cd1	7.62 ± 0.29 ^d^	7.45 ± 0.41 ^d^	2.06 ± 0.01 ^e^
Cd2	14.82 ± 0.74 ^b^	8.89 ± 0.14 ^c^	24.74 ± 0.53 ^a^

Different characters in the column indicate significant difference and the same characters indicate insignificance according to (DMRT) at *p* < 0.05.

**Table 5:** Dry weight tolerance indices for root (TI^r^), shoot (TI^s^) and total plant (TI^tp^) of chickpea seedlings grown in a nutrient medium under Cd stress at the rate of 25 µM (Cd1) and 50 µM (Cd2) with mean values ± SE, (*n* = 3).

Cultivars	Treatments	(TI^r^) ± SE (%)	(TI^s^) ± SE (%)	(TI^tp^) ± SE (%)
NC2	Cd1	74.27 ± 6.41 ^ab^	91.23 ± 0.26 ^a^	87.77 ± 1.43 ^ab^
	Cd2	46.68 ± 5.08 ^bc^	65.53 ± 5.51 ^ab^	61.68 ± 5.40 ^bc^
IC8	Cd1	84.45 ± 2.08 ^a^	95.4 ± 11.37 ^a^	93.21 ± 9.12 ^a^
	Cd2	79.53 ± 18.10 ^a^	86.19 ± 17.23 ^a^	84.85 ± 17.36 ^ab^
IC8-B	Cd1	79.59 ± 9.75 ^a^	92.64 ± 4.55 ^a^	89.77 ± 89.77 ^ab^
	Cd2	29.72 ± 6.36 ^c^	54.72 ± 8.76 ^b^	49.23 ± 8.19 ^c^

Different characters in the column indicate significant difference and the same characters indicate insignificance according to (DMRT) at *p* < 0.05.

**Table 6:** Bioconcentration factor (BCF), bioaccumulation coefficient (BAC) and translocation factor (TF) of chickpea seedlings grown in a nutrient medium under Cd stress at the rate of 0 µM (control), 25 µM (Cd1) and 50 µM (Cd2) with mean values ± SE, (*n* = 3).

Cultivars	Treatments	(BCF) ± SE	(BAC) ± SE	(TF) ± SE
NC2	control	2.16 ± 0.02 ^f^	0.97 ± 0.03 ^d^	0.46 ± 0.02 ^c^
	Cd1	111.41 ± 1.75 ^d^	14.64 ± 0.32 ^b^	0.13 ± 0.01 ^d^
	Cd2	242.81 ± 2.61 ^a^	16.48 ± 1.00 ^ab^	0.07 ± 0.00 ^d^
IC8	control	1.06 ± 0.03 ^f^	1.06 ± 0.01 ^d^	2.01 ± 0.02 ^a^
	Cd1	105.22 ± 0.62 ^d^	14.12 ± 0.13 ^b^	0.13 ± 0.00 ^d^
	Cd2	122.23 ± 1.80 ^c^	18.32 ± 4.54 ^ab^	0.11 ± 0.00 ^d^
IC8-B	control	0.49 ± 0.02 ^f^	0.26 ± 0.03 ^d^	1.06 ± 0.17 ^b^
	Cd1	43.79 ± 0.62 ^e^	21.21 ± 0.17 ^a^	0.48 ± 0.01 ^c^
	Cd2	222.33 ± 6.49 ^b^	8.98 ± 0.06 ^c^	0.04 ± 0.00 ^d^

Different characters in the column indicate significant difference and the same characters indicate insignificance according to (DMRT) at *p* < 0.05.
